# Antioxidant Activities and Anti-Cancer Cell Proliferation Properties of Natsuhaze (*Vaccinium oldhamii* Miq.), Shashanbo (*V. bracteatum* Thunb.) and Blueberry Cultivars

**DOI:** 10.3390/plants2010057

**Published:** 2013-02-15

**Authors:** Hirotoshi Tsuda, Hisato Kunitake, Ryoko Kawasaki-Takaki, Kazuo Nishiyama, Masao Yamasaki, Haruki Komatsu, Chizuko Yukizaki

**Affiliations:** 1 Interdisciplinary Graduate School of Agriculture and Engineering, University of Miyazaki, Miyazaki 889-2192, Japan; E-Mail: vbracteatum@yahoo.co.jp (H.T.); 2 Faculty of Agriculture, University of Miyazaki, Miyazaki 889-2192, Japan; E-Mails: takaki-ryoko@pref.oita.lg.jp (R.K.-T.); nishiyam@cc.miyazaki-u.ac.jp (K.N.); myamasaki@cc.miyazaki-u.ac.jp (M.Y.); 3 School of Agriculture, Tokai University, Kumamoto 869-1404, Japan; E-Mail: hkomatsu@agri.u-tokai.ac.jp (H.K.); 4 Miyazaki Prefectural Food Research and Development Center, Miyazaki 880-0303, Japan; E-Mail: yukizaki@iri.pref.miyazaki.jp (C.Y.)

**Keywords:** anthocyanin, fruit, HL-60 human leukemia cells, polyphenol, *Vaccinium corymbosum*, *V. virgatum*

## Abstract

Antioxidants are abundant in blueberries, and while there are many studies concerning the bioactive compound of fruit, it is only recently that the wild *Vaccinium* species has attracted attention for their diverse and abundant chemical components. The aim of this study was to investigate the bioactive compounds of blueberry cultivars and wild species found in Japan. Among the five extracts of the *Vaccinium* species, Natsuhaze (*Vaccinium oldhamii* Miq.) was found to be the most effective at inhibiting the growth of HL-60 human leukemia cells *in vitro*. Although all ethanol extracts showed a growth inhibitory effect on HL-60 cells, the degree of the effects differed among the species. The extract of Natsuhaze induced apoptotic bodies and nucleosomal DNA fragmentation in the HL-60 cells. Of the extracts tested, that of Natsuhaze contained the largest amount of total polyphenols and showed the greatest antioxidant activity, but the anthocyanin content of Natsuhaze was similar to that of rabbiteye blueberry (*V. virgatum* Ait.). The results showed that total polyphenols contributed to the high antioxidant activity and growth inhibitory effect on HL-60 human leukemia cells of Natsuhaze extract.

## 1. Introduction

There is strong evidence that the antioxidants present in fruits and vegetables protect lipids, proteins and nucleic acids against the oxidative damage initiated by free radicals [1], which play a major role in cancer, heart disease, and vascular and neurodegenerative diseases [2]. Antioxidants are abundantly present in blueberries (genus *Vaccinium*) [3], and numerous studies have been conducted on the bioactive compounds in blueberries and other fruits [4,5]. Although various types of antioxidants have been identified in fruits, anthocyanins and other polyphenols have received the greatest attention [6]. Epidemiological studies showed that some types of cancer are related to dietary habits and that people who consume large amounts of fruits and vegetables have a lower risk of cancer [7]. Fruits of berry species, including blueberries, strawberries, raspberries and cranberries, inhibit multiple stages of carcinogenesis and stimulate the apoptosis of cancer cells [8,9].

**Figure 1:**
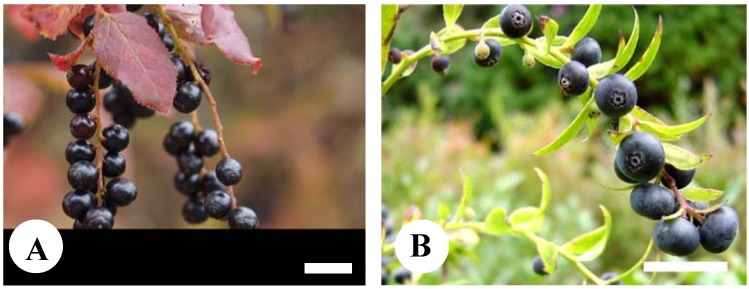
The fruits of Natsuhaze (*V. oldhamii* Miq.) (**A**) and Shashanbo (*V. bracteatum* Thunb.) (**B**). Bars indicate 10 mm.

In Japan, 19 native species of the genus *Vaccinium* are distributed from Hokkaido in the north to the Kyushu region in the south [10]. The deciduous shrub Natsuhaze (*V. oldhamii* Miq.) and the evergreen shrub Shashanbo (*V. bracteatum* Thunb.) (Figure 1) grow naturally in a range from the east to the west of Japan. The edible Natsuhaze and Shashanbo berries are commonly harvested and processed for local foods [11,12]. While the pulp of blueberry cultivars is white, the pulp of Natsuhaze and Shashanbo is tinged with red. Anthocyanins are present in both the peel and pulp of bilberries (*V. myltillus* L.) but mainly in the peel of blueberries [13,14,15]. Therefore, the content of anthocyanins is clearly lower in blueberries than in bilberries on a flesh weight basis [14]. Furthermore, the antioxidant activity is mainly derived from the peel of the blueberry fruit [16], so it is important that anthocyanin and other phenol compounds accumulate in the pulp to raise antioxidant activities of whole fruits. Consequently, it is expected that high amount of anthocyanin with high antioxidant activity in the red pulp of Natsuhaze and Shashanbo. However, the functionality of these two wild *Vaccinium* species has been neither analyzed in detail, nor compared extensively with commercial cultivars of blueberry.

The objectives of the present study were as follows: (1) to determine the total anthocyanin content, total polyphenol content, and antioxidant activity of the fruit extracts of Natsuhaze, Shashanbo and blueberry cultivars; and, (2) to examine the effects of these extracts on the growth and apoptosis of the promyelocytic human leukemia cell line HL-60.

## 2. Results

### 2.1. Anthocyanins

**Figure 2:**
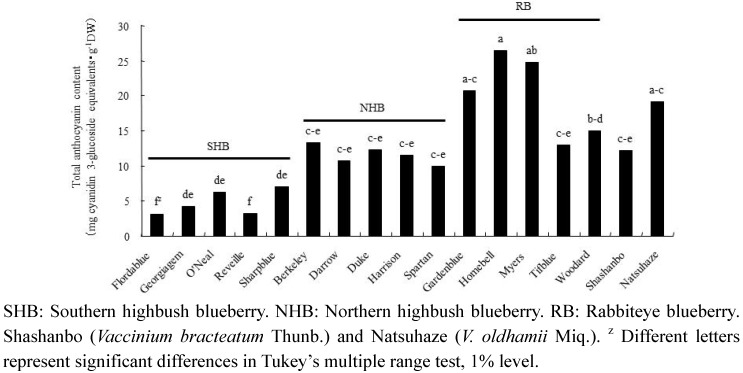
Total anthocyanin content of fruits in blueberry cultivars and wild species.

We detected 14 anthocyanins in all berry extracts. Among the blueberry cultivars, rabbiteye blueberry had a consistently higher total anthocyanin content than the northern and southern highbush blueberries (Figure 2). Although “Homebell” had the highest total anthocyanin content in the fruits (26.5 mg cyanidin 3-glucoside equivalents・g^−1^ DW) among the tested cultivars, it was not significantly different from Natsuhaze (19.1 mg cyanidin 3-glucoside equivalents・g^−1^ DW), “Myers” (24.9 mg cyanidin 3-glucoside equivalents・g^−1^ DW), and “Gardenblue” (20.7 mg cyanidin 3-glucoside equivalents・g^−1^ DW). The anthocyanin profile of extract was also observed at the aglycon level. In northern highbush blueberries, delphinidin compounds constituted the principal anthocyanin, ranging from 39.6% (“Darrow”) to 26.6% (“Duke”), compared to the total anthocyanin. In the southern highbush blueberries, delphinidin or malvidin compounds constituted the principal anthocyanin, ranging from 32.3% (“O’Neal” and “Sharpblue”) to 19.5% (“Gerogeagem”) and 27.8% (“Reveille”) to 24.0% (“O’Neal”), respectively. In the rabbiteye blueberries, malvidin or delphinidin compounds also constituted the principal anthocyanin, ranging from 33.5% (“Homebell”) to 26.2% (“Woodard”) and 28.0% (“Woodard”) to 18.0% (“Tifblue”), respectively. In the wild species, delphinidin compounds constituted the principal anthocyanin, ranging from 32.9% (Shashanbo) to 26.6% (Natsuhaze). Also, the relative content of malvidin was significant in Natsuhaze (20.3%), but Shashanbo contained a large amount of cyanidin compound (31.5%).

### 2.2. Total Polyphenol

**Figure 3:**
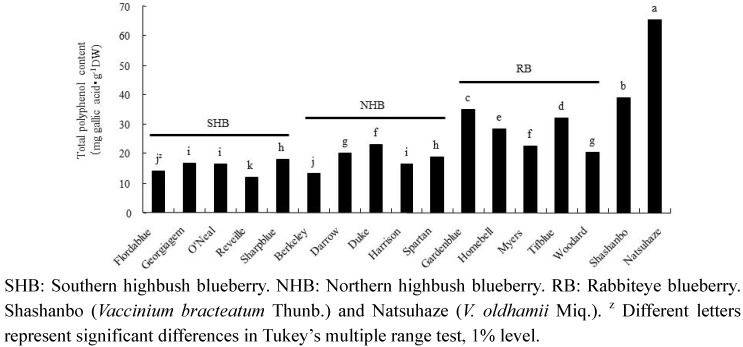
Total polyphenol content of fruits in blueberry cultivars and wild species.

The total polyphenol contents of Natsuhaze (65.5 mg gallic acid・g^−1^ DW) and Shashanbo (38.8 mg gallic acid・g^−1^ DW) were significantly higher than those of the blueberry cultivars (Figure 3). On the other hand, the analysis of the blueberry cultivars indicated that total polyphenol content in rabbiteye blueberry was higher than those of the southern and northern highbush blueberries. Among the blueberry cultivars, “Gardenblue” (35.1 mg gallic acid・g^−1^ DW) was the highest and “Reveille” (12.0 mg gallic acid・g^−1^ DW) was the lowest in total polyphenol content.

### 2.3. Antioxidant Activity

**Figure 4:**
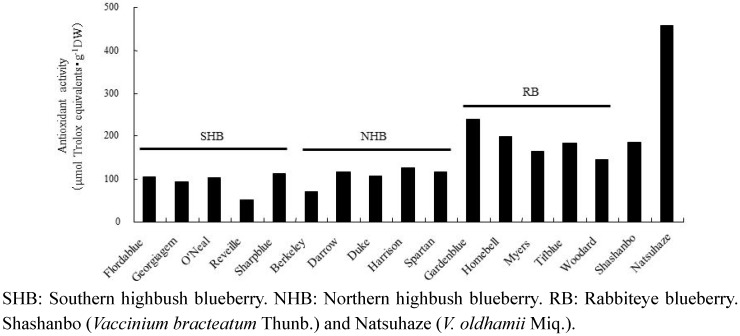
Antioxidant activity of fruits in blueberry cultivars and wild species.

Among the three groups of blueberry cultivars, rabbiteye blueberry showed high antioxidant activity compared to the southern and northern highbush blueberries. Natsuhaze had the highest antioxidant activity in the ethanol extract among all the species analyzed. A large difference in antioxidant activities was observed among the species, ranging from 52.5 (“Reveille”) to 456.2 μmol Trolox equivalents・g^−1^ DW (Natsuhaze) (Figure 4). This represents a > 8-fold difference between the highest and lowest values.

Positive correlations were observed not only between antioxidant activity and total polyphenol content (R^2^ = 0.9283, 17 samples), but between antioxidant activity and the total anthocyanin content (R^2^ = 0.3383, 17 samples).

### 2.4. Growth Inhibitory Effect of Fruit Extracts on HL-60 Cells

**Figure 5:**
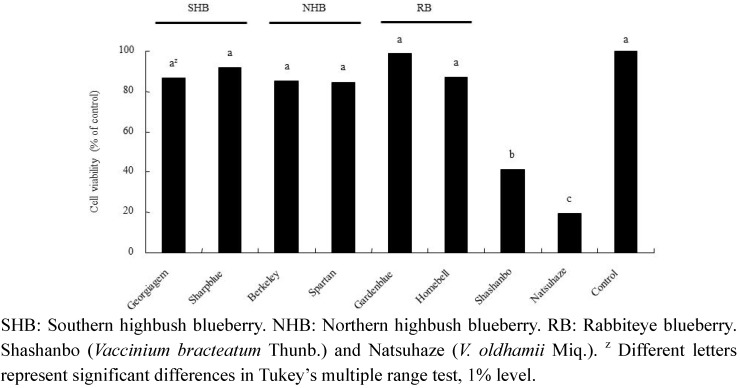
Effect of ethanol extracts on the viability of HL-60 cells.

Differences were observed among the species in their growth inhibitory effect of fruit extracts on HL-60 cells (Figure 5). Natsuhaze and Shashanbo showed significantly higher growth inhibitory effects than the blueberry cultivars, leading to the cell viability of 19.5% and 41.0%, respectively at the same extract concentration (0.5 mg・mL^−1^) (Figure 5). In contrast, blueberry cultivars did not show any significant effect on cell proliferation at the concentration 0.5 mg・mL^−1^ fruit extract.

There was an inverse relationship between the antioxidant activity and cell viability (R^2^* =* 0.5256, eight samples). That is to say, the extracts with the highest antioxidant activity inhibited HL-60 cell proliferation to the highest extent, giving the lowest proliferation levels.

### 2.5. Effect of Fruit Extracts of Natsuhaze on Apoptosis-Induction of HL-60 Cells

**Figure 6:**
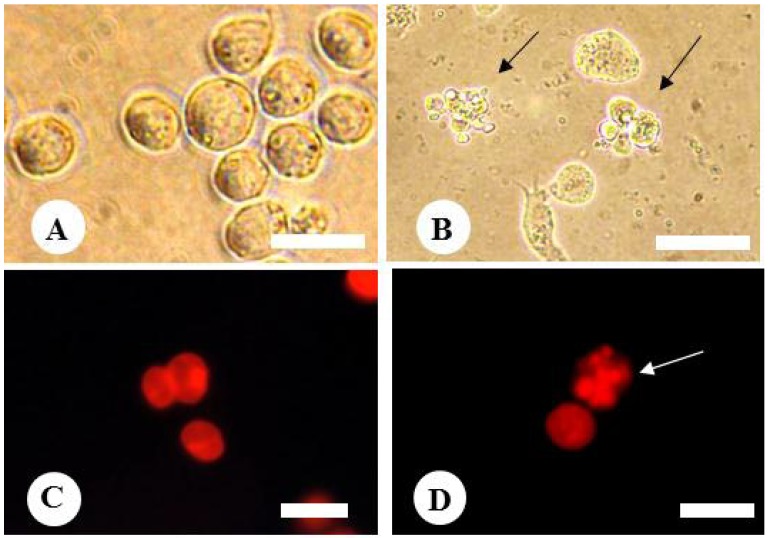
Effect of Natsuhaze ethanol extract on morphological change of HL-60 cells (**A**,**B**) and nuclei (**C**,**D**). (**A**,**C**) Control. (**B**,**D**) HL-60 cells treated with ethanol extract of Natsuhaze at 0.5 mg・mL^−1^. Black arrows indicate the typical apoptotic cells, and white arrow indicates the nuclear condensation. Bars indicates 20 µm (**A**,**B**) and 0.5 µm (**C**,**D**), respectively.

After the 6 h incubation of HL-60 cells with the extract of Natsuhaze at the concentration of 0.5 mg・mL^−1^, apoptotic cell bodies were observed in the cells. The morphological changes of the ethanol extract-treated cells were observed by phase contrast microscopy (Figure 6A,B), and the nuclear condensation of the same cell population was also observed by fluorescence microscopy (Figure 6C,D). These results indicate that the ethanol extract-treated cells were suggesting apoptosis. In contrast, control cells did not show any sign of apoptosis.

**Figure 7:**
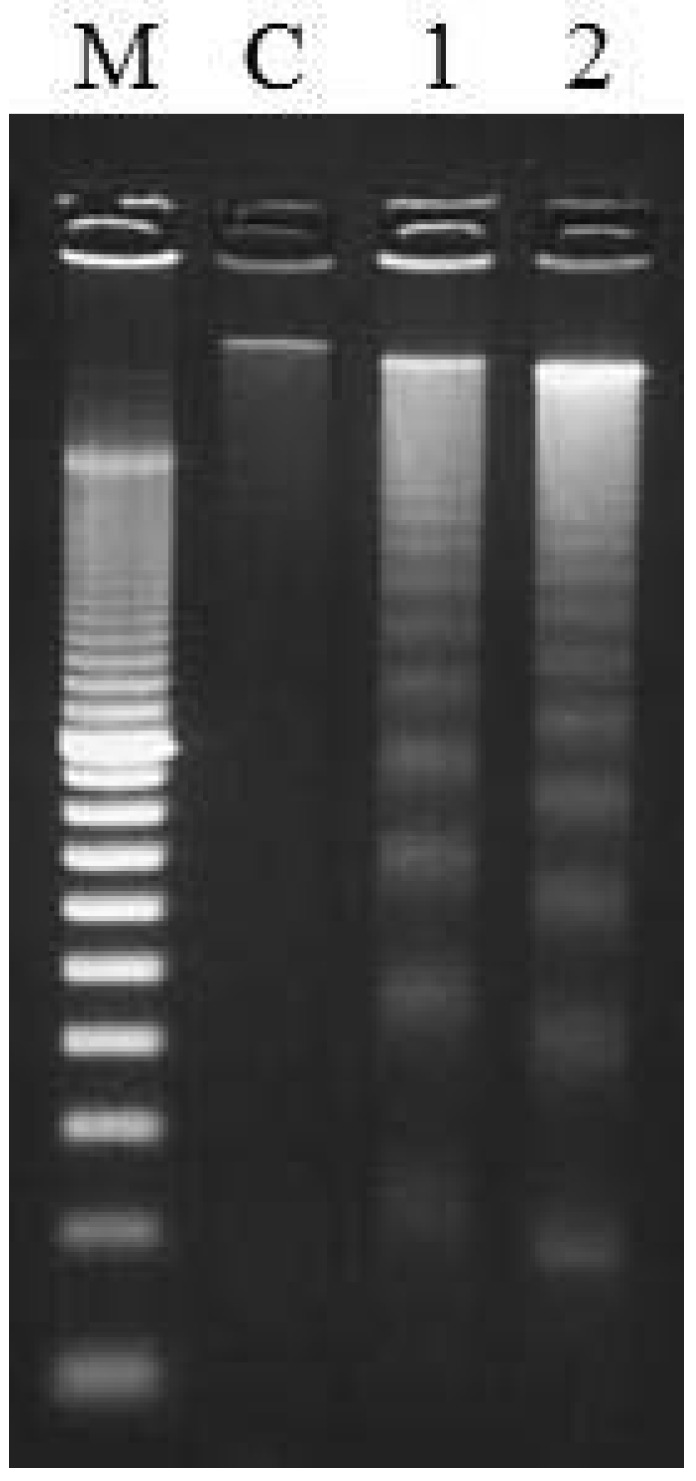
Analysis of DNA fragmentation patterns by agarose gel electrophoresis. DNA was extracted from HL-60 cells incubated with 0.5 mg・mL^−1^ fruit ethanol extract for 6 h. M: DNA marker, C: control, 1: Shashanbo (*Vaccinium bracteatum* Thunb.), 2: Natsuhaze (*V. oldhamii* Miq.).

In addition, the ethanol extract of Natsuhaze induced nucleosomal DNA fragmentation in HL-60 cells at 6 h of incubation (Figure 7). These results indicate that the ethanol extract of Natsuhaze induced apoptosis in HL-60 cells. Similarly, the nucleosomal DNA fragmentation was observed on agarose gel electrophoresis after treatment with Shashanbo ethanol extracts for 6 h.

## 3. Discussion

The health benefits of fruits and vegetables combined in the human diet are well known and are strongly supported by scientific evidence [4]. Fruits and vegetables are also the largest contributors to antioxidant activity, which is considered a dietary quality indicator [17]. Although the antioxidant activity of food is derived from the accumulative and synergistic antioxidant power of vitamins, polyphenols, carotenoids and other minor constituents [18], polyphenols are the main antioxidants present in fruits and vegetables [19]. The production of free radicals in cells and body tissues has been linked to many of the diseases of aging [20,21,22,23]. Polyphenols acting as antioxidants may function as terminators of free radical chains [24]. Therefore, the consumption of fruits with high antioxidant activity can contribute to cancer prevention. Cell culture systems have been used to study the effects of fruit phytochemicals on cancer processes [25]. Consequently, in addition to our analysis of anthocyanin, total polyphenol content and antioxidant activity, we examined the inhibitory effect of fruit extracts on HL-60 cells, using an *in vitro* cell culture system.

In the present study, the anthocyanin contents of Natsuhaze, Shashanbo, and rabbiteye blueberries were not significantly different. On the other hand, we found that the total polyphenol contents of Natsuhaze and Shashanbo were higher than those of the blueberry cultivars. Similar results were reported in another wild species of genus *Vacciniium* [26,27]. The high antioxidant activity of blueberries has been highly correlated to their anthocyanin and total polyphenol contents [26,28]. However, in our present experiment, the total polyphenol contents were strongly correlated with the antioxidant activity. Similar results have been reported by other researchers [27,29], who found a high correlation between antioxidant activity and total polyphenol content. Growth inhibitory effect of extracts in Natsuhaze and Shashanbo on HL-60 cells were significantly higher than those of the blueberry cultivars, and the Natsuhaze extract showed a strong inhibitory effect on the HL-60 cells via apoptosis. The bilberry extract and the anthocyanins, bearing delphinidin or malvidin as the aglycon, inhibit the growth of HL-60 cells through the induction of apoptosis [30]. In our experiments, 0.5 mg・mL^−1^ Natsuhaze extracts significantly inhibited the growth of HL-60 cells at 24 h after being cultured, in contrast, the same concentration of bilberry extract did not significantly inhibit cell growth. Consequently, the growth inhibitory effect of HL-60 cells in Natsuhaze seemed to be higher than bilberry. However, the anthocyanin content of Natsuhaze was lower than rabbiteye blueberry, which was not effective for inhibition of HL-60 cells. Finally, it was thought that the substance which existed in the Natsuhaze extract and inhibited multiplication of HL-60 cells was not anthocyanine. Total polyphenol content of “Gardenblue”, “Homebell”, “Myers”, “Tifblue”, “Woodard” and “Duke” indicated 35.1, 28.5, 22.2, 32.2, 20.4 and 23.0 mg gallic acid・g^−1^ DW, respectively. These were higher than the value (12.8–18.8 mg gallic acid・g^−1^ DW) reported by Wang *et al.* [16] in the same cultivars, which may be attributed to differences in environmental growing conditions. Kalt *et al.* [31] also indicated that synthesis of anthocyanin and other phenolic compounds can be influenced by various abiotic and biotic factors, including temperature, irradiation, herbivory, and pathogenic infection. In any event, the content of total polyphenol was 1.9 to 5.5-fold higher in Natsuhaze (65.6 mg gallic acid・g^−1^ DW) than blueberry cultivars, which may be contributed to the high antioxidant activity and growth inhibitory effect on the HL-60 cells. The antioxidant activity, however, does not explain the high growth inhibitory effect of the Shashanbo extract on HL-60 cells. Other substances in Shashanbo might inhibit the viability of HL-60 cells, but the details are unclear. Generally, antioxidant activity of fruit extract tends to be related to total polyphenol in the fruits [6,32]. The main classes of flavonoids are flavanols (catechins and proanthocyanidins) and anthocyanin, and they contribute antioxidant activity [33,34,35]. The high antioxidant activities of lingonberry (*V. vitis-idaea* L.), cranberry (*V. oxycoccus* L.), and bog blueberry (*V. uliginosum* L.) seem to be related to their high content of catechin or proanthocyanidins, compared to other berries [36,37,38]. Indeed, purified proanthocyanidins from lingonberry have been reported to have very high antioxidant activity in a range of assays [39]. Furthermore, the berry extracts from *Vaccinim* containing a considerable proportion of proanthocyanidins were evaluated for their ability to inhibit growth and stimulate apoptosis of human tumor cells *in vitro* [9,40]. *In vitro* studies employing breast tumor models treated with cranberry extract have reported dose-dependent induction of apoptosis coupled with cell cycle arrest [41]. Treatment of prostate tumor cells (LNCaP) with a wild blueberry proanthocyanidin fraction inhibited the growth of the cells. Moreover, two similar proanthocyanidin-rich fractions from cultivated blueberries significantly inhibited the growth of LNCaP cells [42]. Consequently, the phenolic compounds such as proanthocyanidins in the ethanol extract of Natsuhaze presumably inhibited the growth of HL-60 human leukemia cells through the induction of apoptosis.

## 4. Experimental Section

### 4.1. Fruits

We used two wild *Vaccinium* species, Natsuhaze (*V. oldhamii*) and Shashanbo (*V. bracteatum*), and three types of blueberry cultivars: northern highbush blueberry (*V. corymbosum* L.: “Berkeley”, “Darrow”, “Duke”, “Harrison” and “Spartan”), southern highbush blueberry (*V. corymbosum* interspecific hybrid: “Flordablue”, “Georgiagem”, “O’Neal”, “Reveille” and “Sharpblue”), and rabbiteye blueberry (*V. virgatum* Ait.: “Gardenblue”, “Homebell”, “Myers”, “Tifblue” and “Woodard”). The fruits were harvested in the experimental field of the University of Miyazaki (blueberry cultivars) and at the private nursery in Fukuoka Prefecture (wild species). Each type of fruit was frozen and stored at −30 °C until used for experiments.

### 4.2. Anthocyanins

High-performance liquid chromatography (HPLC) was used to separate and determine individual anthocyanins in the blueberry tissue samples. Frozen berries were extracted with 100% (v/v) methanol containing 1% HCl using a homogenizer (DIAX100, Heidolph, Kelheim, Germany). The homogenized samples were evaporated to dryness, resolublized with pure water, and filled up to 10 mL. These extracts were passed through a 0.22 μm membrane filter (Millipore, Bedford, MA, USA) for HPLC analysis. Samples were analyzed by reversed-phase HPLC using a Shimadzu Prominence LC solution system (Shimadzu, Kyoto, Japan) with a STR ODS-column (Shimadzu, Kyoto, Japan). Chromatographic conditions were as follows: solvent A; 1% (v/v) phosphoric acid; solvent B; 1% (v/v) phosphoric acid, 50% (v/v) methanol and 0.1% (v/v) trifluoroacetic acid; column temperature, 40 °C; detection at 520 nm; flow rate, 1.4 mL・min^−1^. The column was equilibrated with 40% B before use. The binary gradient was as follows: 40%–45% B (0–20 min), 45% B (20–35 min), 50% B (35–45 min), 50%–80% B (45–50 min), 80% B (50–72 min), 40% B (72–80 min). Retention times and spectra were compared with pure standards of malvidin 3-galactoside, peonidine 3-glucoside, cyanidin 3-glucoside, and malvidin 3-glucoside. Other putative anthocyanin peaks were presumed by the method of Ballington [43], and 15 anthocyanins were determined. The results are expressed as mg cyanidin 3-glucoside equivalents・g^−1^ DW. The measurements of sample extracts were replicated three times.

### 4.3. Total Polyphenol

Freeze-dried fruit powders (0.1 g) were dissolved in 80% (v/v) methanol and passed through a 0.45 μm membrane filter (Millipore, Bedford, MA, USA) for analysis. Total polyphenol contents were measured according to the Folin–Ciocalteu reagent method [44]. In brief, 3.2 mL of pure water, 200 μL of each sample, 200 μL of Folin–Ciocalteu’s phenol reagent, and 400 μL of saturated sodium carbonate solution were mixed. The absorbance was read at 760 nm after standing for 30 min. Gallic acid dissolved in 80% (v/v) methanol was used as a standard. The total polyphenol contents are expressed as mg gallic acid equivalents・g^−1^ DW. The measurements of sample extracts were replicated three times.

### 4.4. Antioxidant Activity

The antioxidant activity was determined by an 1,1-diphenyl-2-picrylhydrazyl (DPPH) free radical scavenging assay according to the method described by Suda [45]. In brief, 50 μL of 20% ethanol solution and 50 μL of a 200 mM 2-morpholinoethanesulphonic acid (MES) buffer (pH 6.0) were added to 50 μL of sample solution into a well of a 96-well microplate. The sample solution was prepared by dilution of the fruit extract with 80% ethanol solution. The reaction was initiated by the addition of 50 μL of 1.2 mM DPPH in ethanol. After the reaction mixture had been allowed to stand for 20 min at ambient temperature, its absorbance at 520 nm was measured using an Immuno-Mini NJ-2300 microplate reader (Nalge Nunc International, Tokyo, Japan) with trolox (Aldrich Japan, Tokyo, Japan) as a standard. The antioxidant activity of these extracts is expressed as μmol trolox equivalents・g^−1^ DW. We investigated the correlations between antioxidant activity and the total anthocyanin content, or the total polyphenol content.

### 4.5. Cells and Cell Culture

The HL-60 cell line, human promyelocytic leukemia cells, was originally provided to us by Miyazaki Prefectural Industrial Support Foundation. HL-60 cells were cultured with RPMI-1640 medium supplemented with 10% fetal bovine serum containing 100 units・mL^−1^ penicillin and 100 μg・mL^−1^ streptomycin [46].

### 4.6. Treatment of Cells with Fruit Extracts

Freeze-dried fruit powders (1.0 g) were homogenized in 80% (v/v) ethanol for 30 s. The supernatants were filtered through No. 5 filter paper (ADVANTEC, Tokyo, Japan) and concentrated by rotary evaporation at 37 °C. The extracts were then lyophilized and tested as berry ethanol extracts. A suspension of HL-60 cells (1.0 × 10^5^ cells・mL^−1^) in the culture medium was mixed with ethanol extract and incubated for 24 h. To compare the growth inhibitory effects of the ethanol extracts on the HL-60 cells among the blueberry cultivars and the wild species, the concentrations of these extracts were unified into 0.5 mg・mL^−1^, whereby the concentration indicated the inhibitory effect on HL-60 cells in the wild species based on the results of preliminary experiments. After incubation, cell viability was determined using a cell counting kit-8 (Dojindo, Kumamoto, Japan), which allowed extremely convenient assays with water-soluble tetrazolium salt, WST-8 (2-(2-methoxy- 4-nitrophenyl)-3-(4-nitrophenyl)-5-(2,4-disulfophenyl)-2H-tetrazolium, monosodium salt) that produced a water-soluble formazan dye upon bioreduction in the presence of an electron carrier, 1-methoxy PMS (1-methoxy-5-methylphenazinium methylsulfate). The amount of the formazan produced was directly proportional to the number of living cells. Absorption of the dye corresponding to the cell numbers was measured at 450 nm with the Immuno-Mini NJ-2300 microplate reader. The relative number of surviving cells was estimated in duplicate provided that the value of untreated cells was 100%. The measurements of formazan as the effect of these extracts on the HL-60 calls were replicated five times, and we investigated the correlations between the antioxidant activities and the suppression of cell proliferation.

### 4.7. Determination of Nuclear Morphology

Cells were prepared as described above, and their morphological changes were observed with a phase-contrast microscope (CK40, Olympus, Tokyo, Japan). In the other experiments, morphological changes of nuclei with apoptosis were observed by staining with fluorescent dye. Briefly, the cells which were used in 24 h of the cell proliferation assay were recovered by centrifugal separation (300 × *g*, 5 min). Frozen methanol (−30 °C) was added to the cells on a pellet after two washes with phosphate-buffered saline (PBS), and then the sample was allowed to stand at room temperature for 30 min. Then, the cells obtained by centrifugation (300 × *g*, 5 min) were treated with PBS containing 10 μg・mL^−1^ propidium iodide and 10 μg・mL^−1^ RNase, then incubated at 37 °C for 30 min. The morphological changes of nuclei were observed by fluorescence microscopy (BX51, Olympus).

### 4.8. DNA Extraction and Agarose Gel Electrophoresis

We evaluated the nucleosomal DNA fragmentation by agarose gel electrophoresis [46]. Briefly, cells were washed with ice-cold PBS and lysed with 10 mM Tris-HCl (pH 7.4) containing 10 mM EDTA and 0.5% (v/v) Triton X-100. The cell lysate was treated with 100 μg・mL^−1^ RNase A (Sigma-Aldrich, Steinhein, Germany) at 37 °C for 1 h, followed by 100 μg・mL^−1^ proteinase K (Sigma-Aldrich). Thereafter, DNA was precipitated in 0.5 M NaCl in 50% (v/v) isopropanol and dissolved in Tris-EDTA buffer. The DNA samples were subjected to 2% (w/v) agarose gel electrophoresis, and the nucleosomal DNA fragmentation was detected after SYBR green dye staining.

## 5. Conclusions

Bioactive compounds of blueberry cultivars and wild species of *Vaccinium* in Japan were studied. Among 80% ethanol extracts of five *Vaccinium* species, Natsuhaze (*Vaccinium oldhamii* Miq.) was found to be the most effective at inhibiting the growth of HL-60 human leukemia cells *in vitro*. The ethanol extract of Natsuhaze induced apoptotic bodies and nucleosomal DNA fragmentation in the HL-60 cells. The fruit extracts of Natsuhaze contained the largest amount of total polyphenols and showed the greatest antioxidant activity. The flavonoid content and antioxidant activity of berry crops, including blueberries, are becoming targeted traits by berry breeders [5]. Although considerable variability has been observed in this characteristic, which is quantitatively inherited [47,48], specific breeding for this characteristic has not yet been undertaken [49]. In the present study, we found that Natsuhaze has high antioxidant activity and a growth inhibitory effect of HL-60 cells. Therefore, it could be a useful breeding material for a novel blueberry cultivar with high levels of bioactive compounds.
